# Coordinated Feeding Behavior in *Trichoplax*, an Animal without Synapses

**DOI:** 10.1371/journal.pone.0136098

**Published:** 2015-09-02

**Authors:** Carolyn L. Smith, Natalia Pivovarova, Thomas S. Reese

**Affiliations:** National Institute of Neurological Diseases and Stroke, National Institutes of Health, Bethesda, Maryland, United States of America; UC Irvine, UNITED STATES

## Abstract

*Trichoplax* is a small disk-shaped marine metazoan that adheres to substrates and locomotes by ciliary gliding. Despite having only six cell types and lacking synapses *Trichoplax* coordinates a complex sequence of behaviors culminating in external digestion of algae. We combine live cell imaging with electron microscopy to show how this is accomplished. When *Trichoplax* glides over a patch of algae, its cilia stop beating so it ceases moving. A subset of one of the cell types, lipophils, simultaneously secretes granules whose content rapidly lyses algae. This secretion is accurately targeted, as only lipophils located near algae release granules. The animal pauses while the algal content is ingested, and then resumes gliding. Global control of gliding is coordinated with precise local control of lipophil secretion suggesting the presence of mechanisms for cellular communication and integration.

## Introduction

Placozoans are small disk shaped marine metazoans that attach to a substrate and locomote by ciliary gliding [1]. They stand out from other metazoans in that they lack an internal digestive system and feed by external digestion. They have fewer cell types and a simpler body plan than any other non-parasitic metazoan [2]. *Trichoplax adhaerens* is the sole named member of the phylum but eight distinct haplotypes have been identified in populations collected from the sea [3,4]. Placozoa appear to be less structurally diversified [5] then other early-diverging metazoan phyla (Porifera, Ctenophora and Cnidaria), which have evolved multiple diverse subgroups. The *Trichoplax* genome is small (11,500 genes) and 87% of the genes have homologs in other Metazoa [6]. Recent phylogenetic analyses place the divergence of Ctenophora and Porifera nearest the base of the metazoan tree and Placozoa as sister to the Eumetazoa (Cnidaria and Bilateria), although these placements remain uncertain [7–9]. More detailed information about the functions of the different types of cells in placozoans could provide insight into the evolutionary origins of the cells and organ systems of more complex metazoans.

Information about the cell types and body plan of placozoans comes largely from work on a clone of *Trichoplax* established in the 1970s [10]. A recent structural study on *Trichoplax* based on EM of frozen freeze substituted specimens together with light microscopic techniques provides a more quantitative and detailed view of the cell types and body plan [11]. The animal is bounded by an epithelium, thin on the dorsal side and thick on the ventral side, which attaches to the substrate. The most prevalent cell type in the ventral epithelium is a ciliated epithelial cell responsible for gliding motility. Two types of secretory cells extend processes onto the ventral surface. One is packed with large (~ 0.3 μm) uniform granules, typical of gland cells. These cells are concentrated around the edges of the animal, but also deployed less densely over the entire ventral surface. Gland cells express proteins required for vesicular release, and their vesicles label for neuropeptides. A second type of secretory cell, the newly-discovered lipophil cell, is distributed uniformly in the ventral epithelium. The cell body is located deep in the interior and the entire cell is packed with large granules including one very large granule (~ 3 μm) closely apposed to the ventral surface. Between the dorsal and ventral epithelium is an internal layer consisting of fiber cells with branching processes, the cell bodies of lipophil cells and, arrayed around the edge, crystal cells, each containing a birefringent crystal. The dorsal epithelium in *Trichoplax* consists of flattened ciliated epithelial cells, but another strain of Placozoa collected from the wild has a second type of dorsal cell that, like the lipophil, contains a large, clear granule [11].

Given that *Trichoplax* has been maintained in laboratories for over 40 years, surprisingly little is known about its behavior other than it moves, changes shape and propagates by fission [1,2]. It occasionally pauses and the frequency and duration of pauses increases with the concentration of synthetic food [12]. When feeding on microalgae, *Trichoplax* leaves a trail of lysed algae in its wake and from this it has been inferred that it feeds by external digestion at the ventral surface. However, transepithelial cytophagy by fiber cells has also been inferred based on their cytoplasmic inclusions [13].

Observing feeding *Trichoplax* by light microscopy at scales ranging from the whole animal to subcellular, we find a stereotyped sequence of behaviors beginning with cessation of ciliary beating and gliding, followed by secretion from lipophils of granules whose content, when released, almost immediately lyses algae. Groups of cells then engage in churning movements that may aid in mixing and uptake of material released from the lysed algae. The observation that *Trichoplax* modulates its behavior depending on the presence of food suggests that it has chemosensory cells. The coordinated behavior of cells during feeding bespeaks of mechanisms for intercellular signaling. Two cell types in *Trichoplax* seem possible candidates for controlling its behavior: the gland cells, which contain neuropeptides that could signal in a paracrine manner and the fiber cells, which are interconnected by junctions that may be capable of electrical conduction and which also contact all of the other cell types [11].

## Results

### *Trichoplax* stops movements and ciliary beating in the presence of food

**Fig 1:**
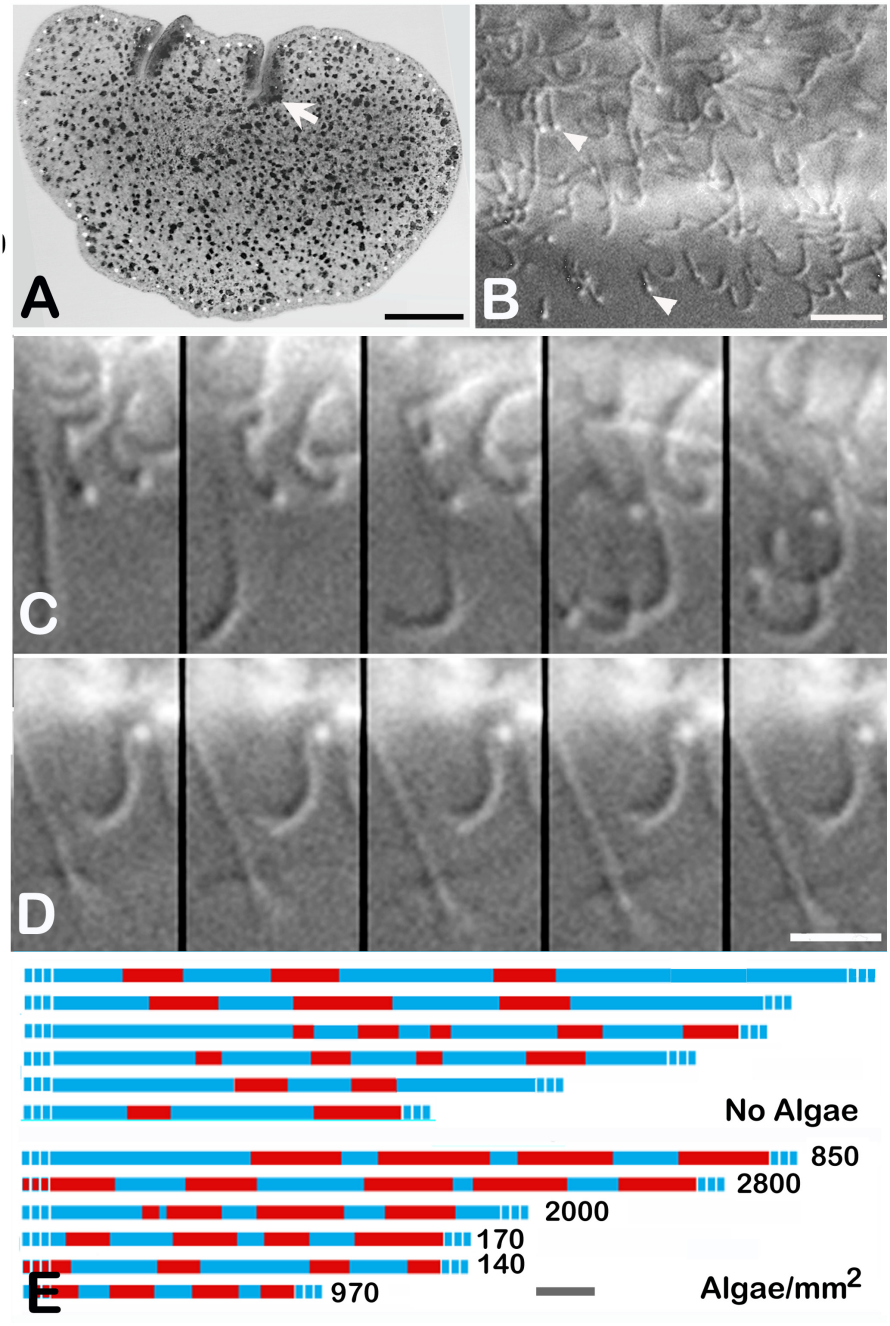
Periodic pauses in motility coincide with cessation of ciliary movements. (A). A *Trichoplax* on a glass substrate viewed with partially polarized transmitted light on a confocal microscope. Invaginations along the edge (arrows) are folds and dark particles throughout the interior are inclusions in fiber cells. The bright particles near the edge are birefringent crystals in crystal cells [11]. (B). Ventral cilia near the edge of a gliding *Trichoplax*. Tips of beating cilia (arrowheads) contact the substrate. The animal was moving toward the bottom of this field. Differential interference contrast (DIC) on a widefield microscope. (C). Five consecutive DIC images (46.5 ms intervals) from the animal illustrated in (B) showing cilia beating asynchronously. (D). Second DIC sequence (46.5 ms interval) during a pause showing arrest of ciliary beat. (E). Time lines track pauses in red and motility between pauses in blue. The top set of lines shows *Trichoplax* in dishes without food. These animals rarely paused (see text; only pausing animals are included in this figure) and those that did pause spend most of the time moving (mean time moving 71%; n = 6). Pauses are more frequent when algae are present (lower set of timelines) and the percent of time moving is smaller (43%; n = 7). Numbers at ends of bars represent concentrations of algae on substrate. Scale bars: A—100 μm; B—10 μm; C-D—5 μm; E—1 min.

*Trichoplax* plated on a glass substrate flattened and adhered to it (Fig 1A). When food was not present, they spent much of the time in various movements on the substrate–migration by gliding, rotation in place, changes in overall shape and folding. The single cilium on individual ventral epithelial cells beat continuously while the animal was gliding (Fig 1B and 1C; S1 Movie). The strokes of adjacent cilia were aligned in the direction of animal’s movement, but the beats were asynchronous. The tips of the cilia appeared to contact the substrate. In migrating animals, the strokes of cilia changed direction coordinately to move the animal in a different direction. Ciliary strokes reversed direction within less than one second during reversals in the direction of rotation (not illustrated).

Some animals moved continually for as long as several hours. Others occasionally ceased moving, some at fairly regular intervals but others less regularly. Pauses began abruptly so cells throughout the entire animal ceased moving within less than a minute (S1 Fig). *Trichoplax* paused more frequently when microalgae (*Rhodamonas salina*; Fig 1E) were present. Ninety percent of animals (n = 30) paused within the first 17 minutes in the presence of microalgae as compared to 22% (n = 76) without food. Animals in the presence of food spent on average 39% of the time pausing (12 animals observed for a total of 5.8 hours) whereas animals without food spent only 2.6% of the time pausing (65 animals observed for a total of 33.5 hours). The difference in percentage of time pausing was highly significant (P< 0.0001, Mann-Whitney, P<0.00004, Kolmogorov-Smirnov). The circumference of the animal typically expanded as the pause began and constricted as the pause ended [12]. When an animal paused, most of the cilia became immobile (Fig 1D), although a few twitched or beat occasionally. As the pause came to an end, cilia gradually resumed beating and the animal gradually resumed gliding, slowly at first and then increasing to the normal speed.

### *Trichoplax* lyse algae during pauses in gliding

The behavior of *Trichoplax* feeding on red microalgae (*Rhodamonas salina*) or green microalgae (*Nannochloropsis*; not illustrated) was monitored by confocal microscopy with transmitted light and fluorescence optics. The transmitted light images revealed the outline of the animal and the locations of cells–fiber and crystal cells–in the interior due to their distinctive inclusions. The microalgae were readily visible by fluorescence microscopy due to the bright fluorescence emitted by pigments in their chloroplasts (chlorophyll and phycoerythrobilin) when excited with 488 or 543 nm illumination [14].

**Fig 2:**
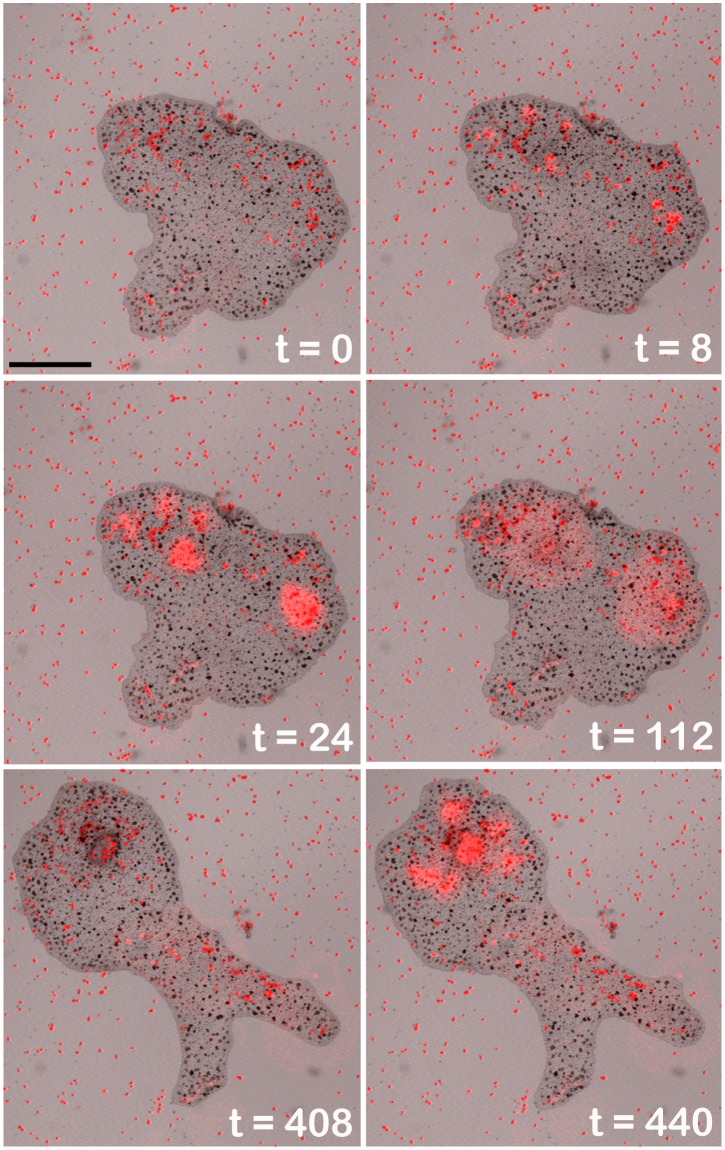
*Trichoplax* feeding on *Rhodamonas salina* microalgae. Algae appear as tiny red specks on the substrate due to their content of fluorescent phycoerythrin. At 8 to 24 sec groups of algae under the paused *Trichoplax* precipitously release their contents apparent as contiguous domains of bright red phycoerythrin. The fluorescence has spread further and diminished at 112 sec. At 408 sec the animal has changed shape and a separate group of algae now covered by the paused *Trichoplax* release their contents at 440 sec. Merged transmitted light and fluorescence images (543 nm illumination) from a confocal microscope. Scale bar—200 μm.

When *Trichoplax* glided onto algae that had settled on the glass surface, their outline stopped changing and cells in the interior became immobile (Fig 2; S2 Movie). Soon after the pause began, the animal lysed underlying algae as evident by the sudden release of fluorescent pigments from the algae. Lysis of microalgae began within 4 to 20 seconds of the beginning of the pause (time lapse interval 4 sec). Clusters rather than widely dispersed algae typically were targeted for lysis. If the animal had multiple algae clusters under its lower surface, it often lysed several of them within a short time (4 to 60 sec for separations up to ~ 1 mm). Some scattered algae outside the clusters might be lysed and while others remained intact.

The fluorescent material released from lysed algae increased in brightness over time, probably due to dequenching of the fluorescence [14]. The material diffused outward from the point of origin over a distance of up to a few hundred microns but remained entirely within the circumference of the *Trichoplax* in all but a few instances until it faded, leaving a faint halo on the cover glass. Cells near the rim of the *Trichoplax* remained stationary during pauses, but cells in more central areas gradually became active, moving along elliptical trajectories (displacement < 200 μm; S1 Fig and S2 Movie), an activity we refer to as *churning*. Animals also formed deep invaginations in their ventral surfaces after lysing algae (see below). The entire animal resumed moving after most of the fluorescent material from the algae faded.

In instances where some intact algae remained on the substrate below the animal, it typically paused again and lysed them. Algae that settled on the dorsal surfaces of *Trichoplax* were not lysed, consistent with the idea that external digestion occurs only at ventral surfaces [2]. Animals typically moved away once most of the algae underneath were lysed. The gliding activity of *Trichoplax* between feeding episodes appeared random, consistent with reports that locomotion in *Trichoplax* is a Brownian type of movement [12].

The behavior during feeding of a second type of placozoan collected from the wild by Florida Aqua Farms (see Methods) was identical to that of the Grell strain of *Trichoplax*. However, unlike *Trichoplax*, these animals also were able to lyse cyanobacteria (not illustrated).

### Lipophil cells secrete granules that lyse algae

Lipophil cell granules in *Trichoplax* were examined during feeding because the spatial distribution and granular content of these cells led us to believe they might secrete digestive enzymes [11]. Lipophil granules were stained with an acidophilic dye, Lysotracker Red, or a lipophilic dye, Lipidtox. The amphiphilic dye FM1-43 was added to the medium because it stained the secreted contents of the granules. High speed two color imaging was performed by spinning disk confocal microscopy (sequential channels 61 ms/ frame) and by confocal microscopy with a resonant scanner (simultaneous two color channels plus a differential interference optics, DIC, transmitted light channel ~150–300 ms/frame).

**Fig 3:**
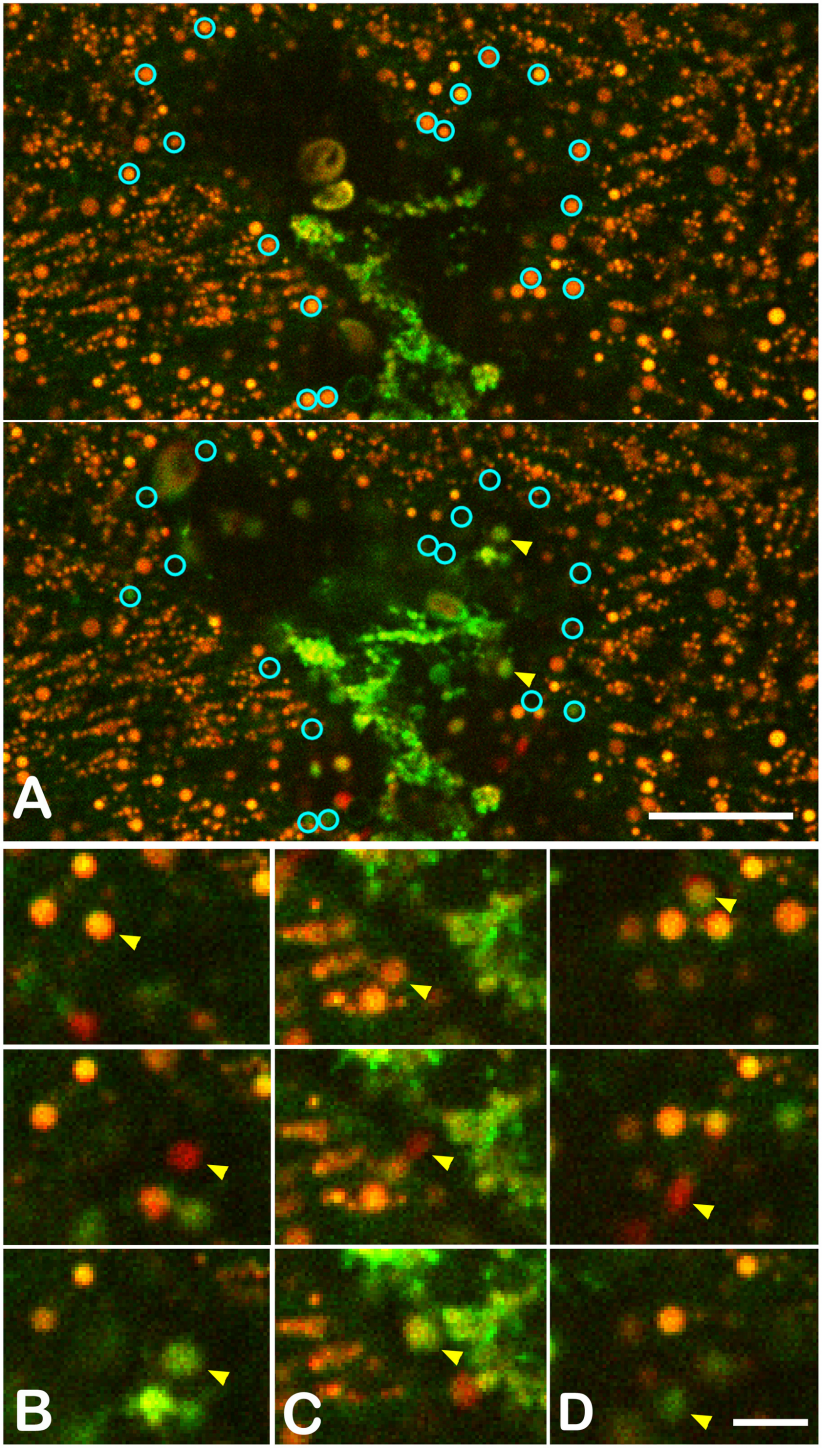
Lipophil granule secretion. (A). Field of lipophil granules (large orange spheres) near a clump of algae and algae debris (yellow-green) prior to and following granule secretion (cyan circles; time interval 308 ms). Secreted granules (yellow arrows) enlarged and became greener due to uptake of FM1-43 dye. (B-D). Sequential images (61.5 ms intervals) showing secretion of individual granules (yellow arrows). Granules appeared as red/green doublets during secretion because the granules were moving and red/green channels were captured sequentially. After secretion, the contents of the granule began to disperse. Lipophil granules were stained with Lysotracker Red (visible in both red and green channels). FM1-43 (green) was added to the seawater to stain the contents of the secreted granules. Sequential two-channel, single focal plane time series collected with a spinning disk confocal microscope. Scale: A- 20 μm, B- D 10 μm.

Images collected near the ventral surface of a *Trichoplax* revealed large ~ 3 μm granules trailed by chains of deeper and smaller granules that appeared orange with the spinning disk system (Fig 3A and S3 Movie) and red by scanning confocal microscopy (S4 Movie). Granules were essentially immobile in paused animals. Granule secretion occurred in the vicinity of algae and was marked by abrupt movement or disappearance of one or more large orange/red lipophil granules (Fig 3B–3D and S3 Movie). Following the secretion of the granule, a burst of green (FM1-43) fluorescence usually was visible in the place that the granule was last seen. The green fluorescence usually diffused away rapidly, but in a few instances a ball of green fluorescence persisted for several seconds.

**Fig 4:**
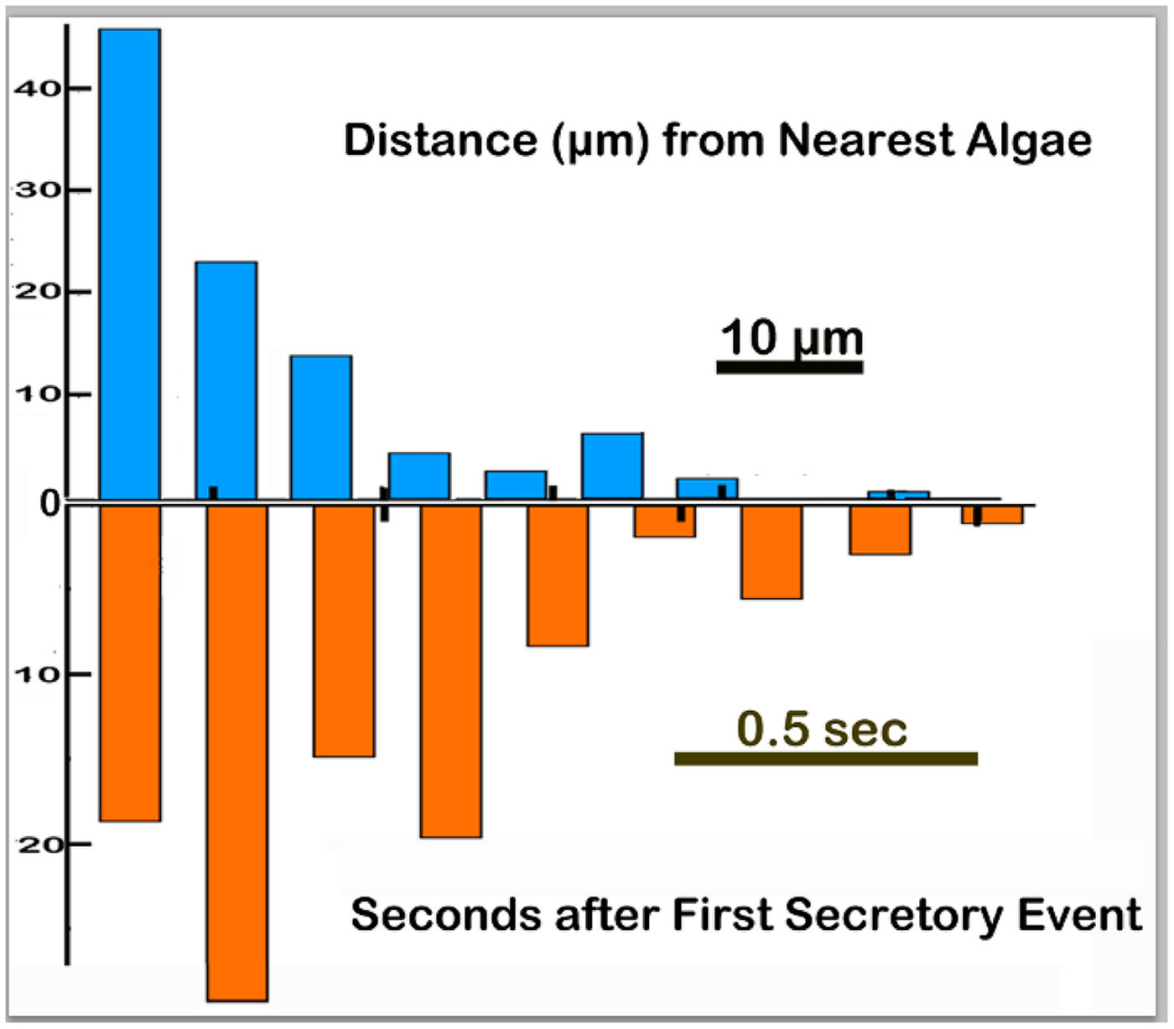
Frequency histograms of measurements of the timing (red) and positioning (blue) of individual secretory events as evident by the abrupt extrusion of a lipophil granule. Time measurements, in seconds, started when the first granule was released (N = 107 granules from eight time lapse sequences). Distance measurements show how far in microns each granule was from the nearest fluorescent algal cell or algae debris.

Lipophils that secreted granules typically were located within 15 μm of the closest algae (Fig 4 top graph). When only a few scattered algae were present under the region of the *Trichoplax* in the field of view (~200 x 200 μm), only few granules in the near vicinity of the algae were released (not illustrated). However, when multiple algae were present, many more granules were secreted. Group secretory events typically began with secretion from a few lipophils adjacent to an algae followed within less than ~ 1.5 seconds by secretion from multiple lipophils spread over a wider area (Fig 4 bottom graph and S3 Movie 1 and 2). The algae began to grow brighter within one second of secretion and dispersal of the granule, indicating that lysis was releasing algal contents (S4 Movie). The microalgae often jumped long distances (>20 micron) during lysis, probably due to activation of the ejectosome, an escape device in cryptamonad algae [15]. Lipophils secreted only their large ventral granule. Smaller granules deeper inside were visible but not secreted. We assume that the secreted granule eventually was replaced with another large granule but this never occurred within the several minutes during which the animal was sufficiently stationary to track the positions of lipophil cells.

When multiple lipophils secreted granules, the released liquid was visible by DIC imaging (not illustrated) because its refractive index differed from the surrounding seawater. The area of the ventral surface overlying secreted granules rapidly moved further from the glass surface, perhaps due to displacement by the discharged material. *Trichoplax* feeding on algae leave an apparently sticky substance along their path of movement upon which particulate matter collects (not illustrated), along with undigested remnants of algae, suggesting that the secretions of lipophils may include a mucous-like substance.

Fluorescent material continued to diffuse out of lysed algae for ~ 1 minute, and some of the material stuck to the surface of the *Trichoplax* (S4 Movie). It was during these times that animals exhibited the churning behavior referred to above and, in many instances, formed deep folds in their ventral surfaces. The fluorescent material on the surface gradually disappeared over the course of minutes.

### Lipophils exhibit characteristics of secretory cells

**Fig 5:**
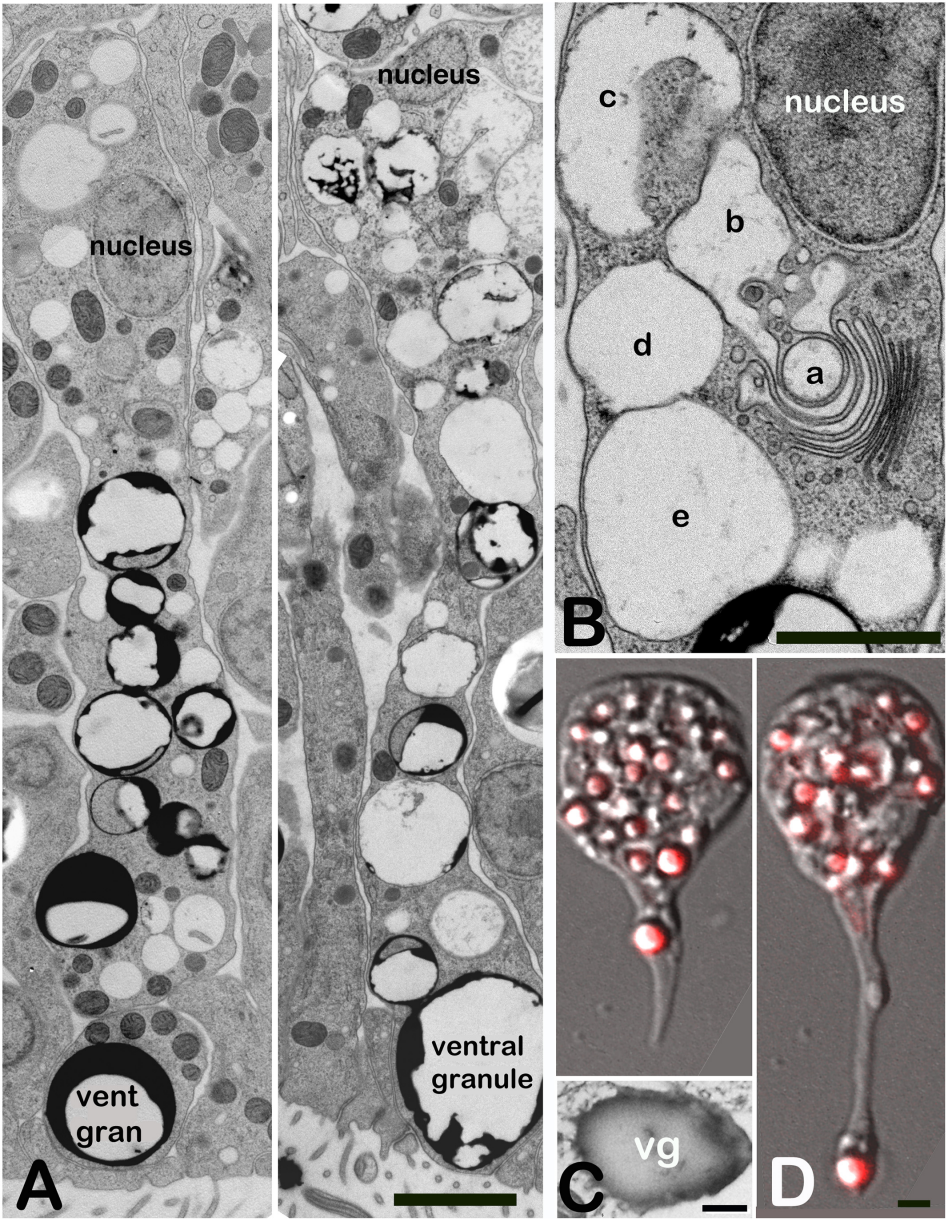
Lipophil cell inclusions. (A). Electron micrographs of long axes along lipophil cells prepared by freezing and freeze substitution. They are packed with extending granules from the cell bodies (nucleus) to the ventral surface where a large ventral granule lies closely apposed to the plasma membrane. Granules vary in their contents throughout the lipophil. (B). Deep in the cell body (nucleus) granules are produced by the Golgi apparatus (lettering indicates probable order). (C). Unfixed frozen section through a lipophil granule shows that these granules actually have uniformly dense contents. (D). Live cell imaging of a lipophil isolated from a *Trichoplax* showing that granules are transported from the cell body anterogradely down processes. Scale bars: A B, D—2 μm, Scale bar C -1 μm.

The large granules secreted by lipophils during feeding corresponded in size and location to the large ventral granules (2 to 3 μm) in lipophil cells of *Trichoplax* prepared by freeze substitution and visualized by TEM (Fig 5A). These large granules were closely apposed to the ventral surface, typically separated from it by10 to 20 nm. Lipophil cells extended deep into the interior of *Trichoplax* and were packed with smaller granules along the entire longitudinal extent of the lipophil. Granules were membrane bound and some of the granules in the cell body were intimately associated with the trans region of the Golgi complex, which was located near the nucleus (Fig 5B). Vesicles in lipophils never appeared fused with each other. The granule contents in freeze-substituted lipophils varied in electron opacity, but appeared uniformly dark gray in frozen cryo-sectioned material (Fig 5C and S2 Fig). Electron probe analysis of their granular content further differentiated lipophil granules by revealing a high level of sulfur compared to the small, darker granules of ciliated ventral epithelial cells which contained high levels of chloride instead (S2 Fig).

The lipophil cells in preparations of dissociated *Trichoplax* cells were readily identifiable due to their content of granules that appeared refractile by DIC optics and by their uptake of Lysotracker Red (Fig 5D). Some disassociated lipophils contained both large and smaller granules, others only smaller granules, possibly because the large granules were discharged during dissociation. Isolated lipophils were rounded or elongated, with a rounded cell body and a narrow process. If an elongated cell contained a large granule, it typically was located in the narrow process. Time lapse imaging showed that the lipophil granules were in constant motion. Both large and smaller granules were transported anterogradely and retrogradely within the processes of elongated lipophil cells. The processes of some lipophil cells were motile, elongating, retracting, and bending.

## Discussion

*Trichoplax* has a surprisingly complex and well-coordinated behavioral repertoire for an animal with only six cell types and apparently no nervous system [2,11]. The animal’s gliding movements are propelled by asynchronously beating cilia that can rapidly reorient their strokes to move the animal in a different direction. Cilia cease beating and gliding stops when the animal pauses to feed. Specialized secretory cells-the lipophils-secrete large ventral granules whose content promptly lyses nearby algae. Groups of lipophils, even ones widely separated in the animal, secrete almost simultaneously. Groups of cells in the center of the animal begin to make elliptical churning movements while the animal is paused and material from the lysed algae is ingested, likely by ciliated ventral epithelial cells [16]. The ciliary beat resumes and the animal moves on only after most of the material released from the algae has disappeared. We propose that the remarkable spatial and temporal coordination of cellular activities during locomotion and feeding must require both local and long range signaling and control systems.

When *Trichoplax* is not feeding, it is migrating or rotating in place. Both migration and rotation are propelled by asynchronously beating cilia whose tips appear to achieve traction with the substrate. The strokes of the cilia align with the direction of the animal’s movement and the cilia can rapidly change or reverse the direction of their strokes to reverse the direction of gliding. Although ciliary gliding occurs in several groups of animals, that found in *Trichoplax* appears to be novel. Cilia in cnidarian larvae, annelids, and planarians instead beat metachronously during gliding [17–19], as do cilia used for swimming [20]. Cells in epithelia generally are interconnected by gap junctions that provide for electrical signaling, movement of small molecules, and coordination of cilia [21] but no gap junctions or genes associated with gap junctions are present in *Trichoplax* [2,6,11].

A key finding by live cell microscopy is that lipophil cells secrete granules whose contents lyse algae, thus identifying lipophils as principle digestive cells in *Trichoplax*. TEM images of lipophils show that they contain large membrane limited vesicles cued up continuously back to the Golgi apparatus located deep in the interior adjacent to the nucleus. Anterograde and retrograde transport of granules in living lipophils is apparent by light microscopy. The ventral most granules in lipophils in the intact animal are larger than ones further back in the cell, perhaps reflecting a final step in readying their contents for release. Lipophil granules appear mostly empty after either typical chemical fixation or freeze substitution [11] but direct examination in frozen sections shows that they have an electron dense content that includes high concentrations of sulfur. This distinguishes them from granules in adjacent cells and perhaps signals the presence of sulfomucins typical of some types of secretory cells [22].

The precision with which lipophils secrete granules to lyse underlying algae appears to require chemosensory cells to detect algae as well as way to couple sensory cell activation with lipophil secretion. Chemosensory cells typically have either a cilium or microvilli [23], which lipophils lack. Gland cells, however, present both a cilium and microvilli. Moreover, gland cells express proteins required for fast, regulated secretion (syntaxin 1/2, synaptobrevin, SNAP25 and synapsin] and package neuropeptides [11] and possibly other neurotransmitters [6,24] that might activate lipophil secretion by a paracrine signaling mechanism.

Gland cells comprise about 3% of the total cell population in the ventral epithelium [11]. Approximately half are concentrated in a ring about 20 nm from the edge, while the rest are interspersed inside, among the other ciliated ventral epithelial cells and lipophils. Only the gland cells interspersed inside are appropriately positioned to sense algae and target lipophil secretion by paracrine signaling. Global signaling mechanisms appear to be required to explain the cessation of ciliary beating that precedes feeding and the almost simultaneous secretion by separate groups of lipophils. The gland cells that are arrayed around the edge are possible candidates for secreting a signal that could diffuse across the entire animal. The observation that gland cells around the edge label for FMRFamide, whereas those in the interior do not [11,25] supports the idea that the two populations of gland cells may have different functions.

Cells near the edge of the animal remain stationary during a feeding episode, but more centrally located cells begin churning movements soon after lysis of algae. The churning movements involve cells in the ventral epithelium, evident by observing lipophil granules stained with fluorescent dyes, as well as fiber cells in the interior, visible by transmitted light imaging due to their content of opaque inclusions. The churning movements are slow and undulatory, in this respect bearing a resemblance to movements in smooth muscle or sheets of myoepithelial cells. Although myofibrils have not been detected in any cell type in *Trichoplax*, it has been suggested that fiber cells may be contractile [2,26]. Fiber cells have transverse branches that contact other fiber cells and the cell bodies of lipophils, ventrally directed branches that penetrate deep into the ventral epithelium and branches that contact dorsal epithelial cells [11]. Moreover, fiber cells are interconnected to a least some extent by junctions that resemble the electrically conductive syncytial junctions of glass sponges [11,27–29]. Whether fiber cells are indeed electrically excitable and contractile and if so how they might be activated during feeding are questions remaining to be addressed.

In respect to design and functions the ventral epithelium of *Trichoplax* has similarities to the walls of the digestive tracts of mammals which include, in addition to multiple types of cells that secrete digestive enzymes and mucous, enteroendocrine cells thought to be chemosensory for bitter and sweet compounds as well as amino acids [30]. These chemosensory enteroendocrine cells secrete peptides that control digestive tract secretion and motility as well as behaviors associated with food ingestion. Surrounding the digestive tract are muscles that control passage of material through the tract. Although *Trichoplax* lacks muscles, it has a contractile apparatus to produce churning movements during feeding.

## Conclusions

Placozoa are unique among extant animals in feeding by external digestion. We show that in *Trichoplax*, as well as another type of placozoan, that this feeding strategy employs complex control mechanisms. Global control is needed to pause over food while digesting it and to coordinate lipophil secretion across the animal, and precise local control is needed to target secretion of digestive enzymes onto individual clumps of algae right underneath. *Trichoplax* does not have synapses but does have gland cells containing peptides and typical secretion machinery that might participate in the control of feeding. Fossil animals found in Ediacarian sediments appear to have flattened body plans and to have moved and left imprints on algal mats [31–35]. Some of these, like Trichoplax, may have lacked a gut and digested algae externally. Achieving a detailed mechanistic understanding of external digestion in a modern animal may provide a window to understanding the early evolution of digestion and the systems controlling it.

## Materials and Methods

### Animals

*Trichoplax adhaerens* of the Grell (1971) strain, gift of Leo Buss (Yale University), were maintained in culture with *Rhodamonas salina* algae (also called *Pyrenomonas salina*, Bigelow National Center for Culture of Marine Algae and Microbiota, East Boothbay, ME) as described previously [11,36]. Placozoans we discovered in a shipment of marine sponges collected from the wild (Florida Aqua Farms, Dade City, FL) also were examined. These placozoans were maintained in a saltwater aquarium containing self-sustaining phytoplankton (mainly cyanobacteria) without added food. *Trichoplax adhaerens* (Grell) were used for all measurements and illustrations.

### Light microscopy

Prior to imaging animals were transferred to a Lab-Tek II cover glass chamber (Lab-Tek, Rochester, NY) or Warner RC-40LP chamber with #1.5 cover glass (Warner Instruments, Hamden, CT) containing filtered seawater. Images of whole animals were collected on a LSM510 confocal microscope (Carl Zeiss Microscopy LLC, Thornwood, NY) with a 10X 0.45 NA or 5X 0.25 NA objective. Transmitted light images were collected with 633 nm illumination (Fig 1 and S1 Fig). Combined transmitted and fluorescence images were with 543 nm illumination and a 560 long pass filter (Fig 2 and S2 Movie). Images of cilia (Fig 1, S1 Movie) were captured at 7.6 ms/frame with an Orca 4.0 CMOS camera (Hamamatsu Photonics) on an Olympus microscope (Olympus Corporation, Waltham, MA) with 60X NA 1.45 objective, differential interference contrast (DIC) optics and Metamorph image acquisition software (Molecular Devices, Sunnyvale, CA).

Lipophil granules were stained with 0.3 μM Lysotracker Red or Lipidtox and 0.75 μM FM1-43 (Invitrogen, Carlsbad, CA). High speed fluorescence images were collected on a spinning disk confocal microscope (Nikon Ti-E, Nikon Instruments) with 40X 1.3 NA objective, sequential 488 and 561 nm illumination and dual 503–530, 580–620 filter (Fig 3, S3 Movie) or on a resonant scanner confocal microscope (Leica SP5, Leica Microsystems, Mannheim, Germany) with 40X 1.2 NA objective, 488 and 633 nm illumination and 500–600 and 640–750 detection windows (S4 Movie). Merged DIC and fluorescent images of dissociated lipophils were collected with a Zeiss LSM510, 63X NA 1.4 objective, and 543 nm illumination and 560LP filter (Fig 5D).

Dissociated cells were prepared by incubating animals in calcium and magnesium-free seawater with 0.25% trypsin at room temperature for 45–60 min. The animals were transferred into seawater and broken apart by trituration with a fire-polished glass pipette.

### Electron microscopy

#### Freeze-substitution and transmission electron microscopy

*Trichoplax* were prepared by freeze-substitution exactly as previously described [11]. Briefly, animals were transferred to specimen carriers (Leica, Bannockburn, IL) and high pressure frozen in a Baltec 010 high pressure freezing machine (TechnoTrade International, Manchester, NH). Specimens were slowly warmed in a Leica AFS1 freeze-substitution machine (Leica, Bannockburn, IL) in dry acetone contain uranyl acetate, osmium tetroxide, and hafnium chloride. Embedding was in Epon and sections ~70 nm thick were photographed in a JEOL 200-CX electron microscope (Jeol USA, Inc., Waterford, VA).

#### Cryosectioning and x-ray microanalysis

*Trichoplax* were frozen and cryosectioned as previously described for isolated frog ganglia [37]. Briefly, *Trichoplax* in artificial sea water were mounted on agar/gelatin pads and rapidly frozen against a liquid nitrogen cooled copper block (LifeCell CF-100, The Woodlands, TX). Cryosections ~80 nm thick were cut with a Leica UC6 cryo-ultramicrotome (Leica, Bannockburn, IL) at 160°C. Sections mounted on Formvar/carbon-coated grids were cryotransferred into an EM912 Omega electron microscope (Carl Zeiss Microscopy, Thornwood, NY) and freeze-dried. X-ray spectra were recorded as previously described [33]. Concentrations of Na, P, S, Cl, K and Ca are mmol/kg dry weight.

## Supporting Information

S1 Fig Kymograph of gliding *Trichoplax* showing a single pause lasting ~ 4 min. The diameter of the animal constricts at the beginning of the pause (between red arrows) and expands at the end of the pause. This kymograph shows a time series of narrow windows 10 μm wide normal to the axis of crawling so stationary objects appear as horizontal lines where cells inside are stationary. Many lines of stationary cells are evident during the pause, but some cells begin to move near the end of the pause (blue arrow) as evident from the vertical deviations of the lines. Scale bars: 20 μm (vertical) and 40 sec (horizontal).
(TIF)

S2 Fig Cryosections of directly frozen *Trichoplax*. (A). Section deep inside the *Trichoplax* passes through cell body of lipophil cell (nucleus at N) showing that secretory granules have dense contents in the cell body as well as in the ventral process. (B). Ventral epithelial cell near ventral surface (ciliary root at green arrow). contains clusters of smaller granules with dense content. (C). X-ray spectra from lipophil and ventral epithelial cell granules (VEC) show a marked difference in their contents of sulfur and chloride. Scale bar 1.0 μm.
(TIF)

S1 Movie Asynchronous beating of *Trichoplax* cilia during gliding motility. Time series captured with differential interference optics on a widefield microscope. Scale bar (first image)-10 microns. Time shown at lower left.
(MP4)

S2 Movie *Trichoplax* feeding on *Rhodamonas salina* algae. The animal pauses twice, each time lysing algae (small red particles), evident by the release of phycoerythrin (red clouds). After each lytic event, groups of cells in the interior begin churning motions (~ 80 sec and ~500 sec), evident by the elliptical movements of fiber cell inclusions (dark particles). Fig 2 shows representative images from this series. Merged fluorescence and transmitted light (543 nm) on a confocal microscope.
(MP4)

S3 Movie Lipophil granule secreting contents. At the beginning of the sequence, the large ventral granules and smaller interior granules of lipophil cells (orange spheres) are immobile in an animal that has paused over a clump of algae and debris (green-yellow). At 33.4 seconds (time at upper right), scattered lipophil cells in the vicinity of the algae secrete their large ventral granules. Colors of moving granules are not super-imposed due to sequential channel acquisition. The granule content becomes greener after release due to uptake of FM1-43. The secreted content begins to diffuse away instantly or within a few seconds. Lipophil granules stained with Lysotracker Red; FM1-43 in bath stains released content of granules. Single focal plane time series from a spinning disk confocal microscope with 488 and 561 nm illumination. Field of view-180 x 180 μm.
(MP4)

S4 Movie Algae lysis is followed by folding and churning. Large ventral granules of lipophil cells (small red particles) interspersed among other ventral cells (dim green background) are stationary in an animal paused over several clumps of algae (large orange ellipses) and debris (bright green). Lipophil cells in the vicinity of algae secrete granules at 18:04:06 to 18:04:07 (time at upper right). Algae begin to turn yellow-green shortly after granule release indicating that they have lysed. Algae continue to release green material, some of which sticks to the surface of the ventral cells, for the duration of the sequence. During this time, cells in the vicinity of the algae churn and the ventral surface develops deep invaginations (18:04:10). Lipophil granules stained with Lipidtox; FM1-43 stains plasma membrane (dim) and secreted contents of granules (brighter). Single focal plane, simultaneous red/green channel times series from high speed scanning confocal microscope with 488 and 633 nm illumination. Field of view 230 X 230 μm.
(MP4)
