# Supplementary figures and images for: Coordinated Feeding Behavior in Trichoplax, an Animal without Synapses
Source: PLoS One. 2015 Sep 2;10(9):e0136098. doi: 10.1371/journal.pone.0136098 (PMC4558020; doi:10.1371/journal.pone.0136098)

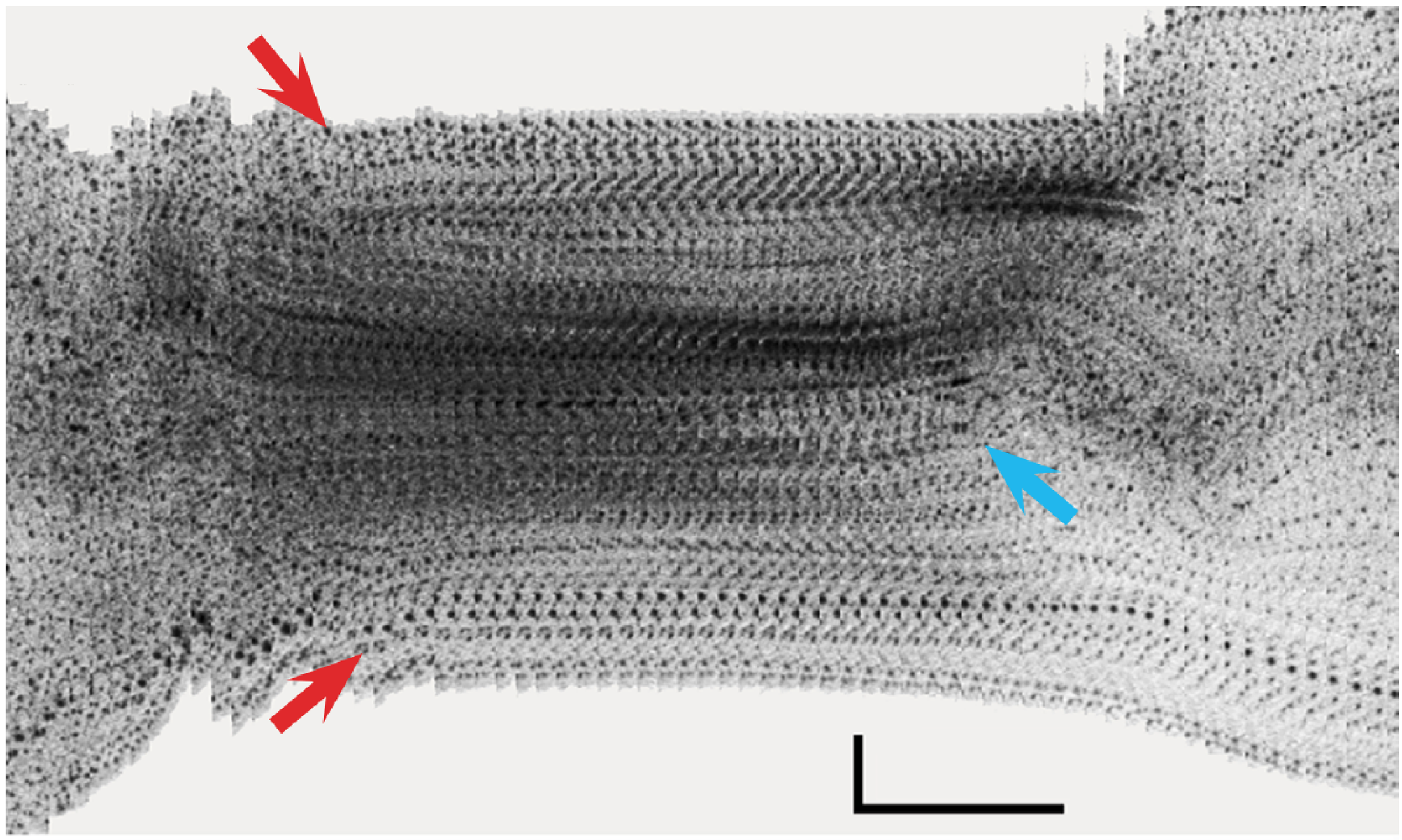

Supplement: S1 Fig — The diameter of the animal constricts at the beginning of the pause (between red arrows) and expands at the end of the pause. This kymograph shows a time series of narrow windows 10 μm wide normal to the axis of crawling so stationary objects appear as horizontal lines where cells inside are stationary. Many lines of stationary cells are evident during the pause, but some cells begin to move near the end of the pause (blue arrow) as evident from the vertical deviations of the lines. Scale bars: 20 μm (vertical) and 40 sec (horizontal). (TIF) [file pone.0136098.s001.tif]

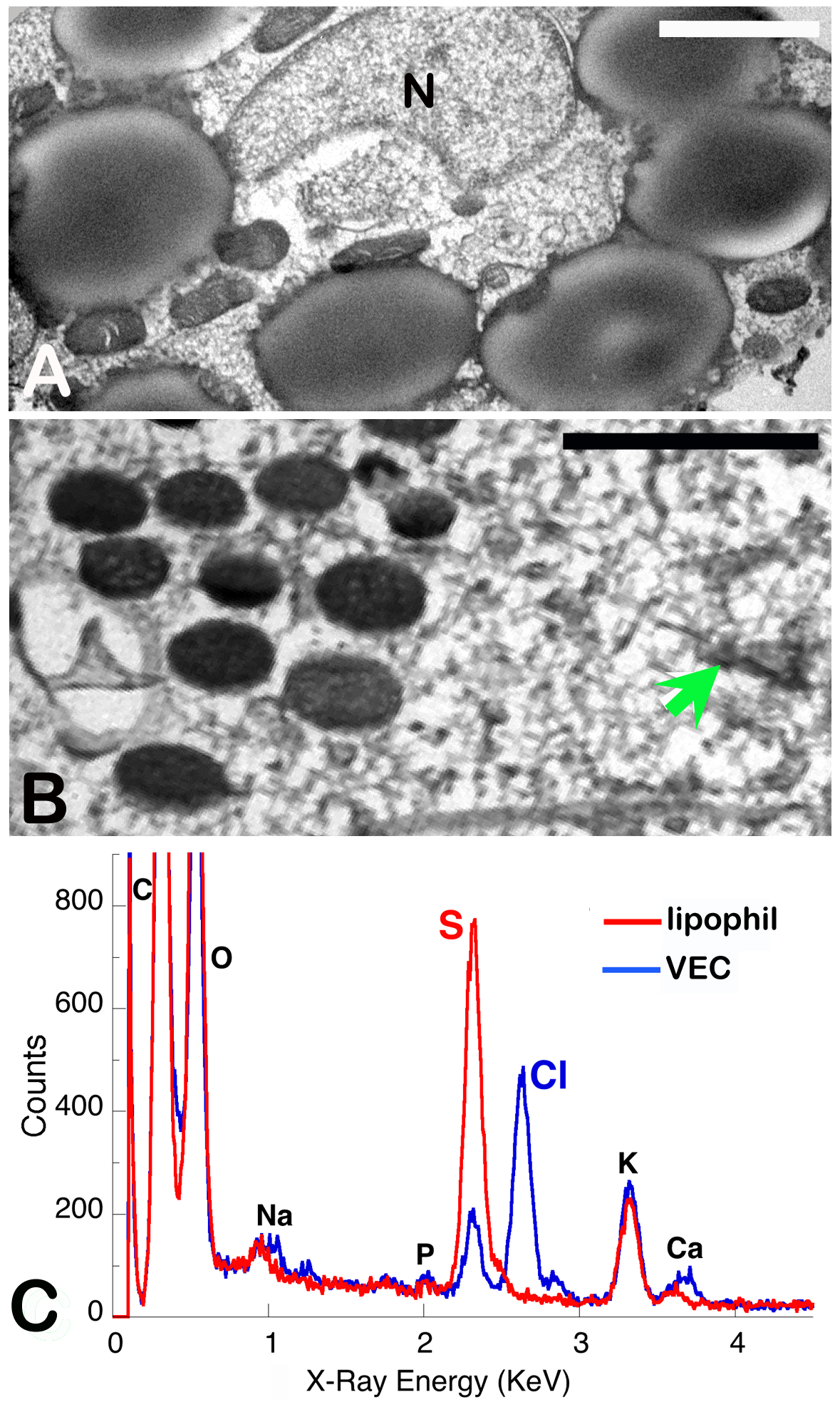

Supplement: S2 Fig — (A). Section deep inside the Trichoplax passes through cell body of lipophil cell (nucleus at N) showing that secretory granules have dense contents in the cell body as well as in the ventral process. (B). Ventral epithelial cell near ventral surface (ciliary root at green arrow). contains clusters of smaller granules with dense content. (C). X-ray spectra from lipophil and ventral epithelial cell granules (VEC) show a marked difference in their contents of sulfur and chloride. Scale bar 1.0 μm. (TIF) [file pone.0136098.s002.tif]
